# Impact of the addition of Early Embryo Viability Assessment to morphological evaluation on the accuracy of embryo selection on day 3 or day 5: a retrospective analysis

**DOI:** 10.1186/s13048-019-0547-8

**Published:** 2019-08-09

**Authors:** Alberto Revelli, Stefano Canosa, Andrea Carosso, Claudia Filippini, Carlotta Paschero, Gianluca Gennarelli, Luisa Delle Piane, Chiara Benedetto

**Affiliations:** 1 Department of Surgical Sciences, Gynecology and Obstetrics 1, Physiopathology of Reproduction and IVF Unit, S. Anna Hospital, University of Torino, Via Ventimiglia 3, 10126 Torino, Italy; 2 Department of Surgical Sciences, Clinical statistics, University of Torino, Corso Bramante, 88 Torino, Italy

**Keywords:** Time lapse, Embryo score, IVF, Clinical pregnancy rate, Ongoing pregnancy rate

## Abstract

In this study we aimed at retrospectively assessing in a homogeneous group of IVF patients whether the addition of Early Embryo Viability Assessment (Eeva™) to standard morphology increases the accuracy of embryo selection in case of double embryo transfer (DET) on day 3 or single embryo transfer (SET) on day 5. Eeva™ is an algorhythm aimed at indicating on day 3, according to morphokinetic parameters observed in the first three days of embryo growth, which embryos are more likely to develop into viable blastocysts and implant. A total number of 328 patients were included in the study; IVF or ICSI were performed and 428 embryos were transferred, either with DET on day 5, or (when at least four top scored embryos were available on day 3) with SET of day 5. Four groups were considered: (a) patients receiving day 3 DET with embryos selected by standard morphology (DET-3 M, *n* = 106, receiving 212 embryos), (b) patients receiving day 3 DET with embryos selected by morphology plus Eeva™ (DET-3 ME group, *n* = 48, receiving 96 embryos), (c) patients receiving day 5 SET with a blastocyst selected by standard morphology (SET-5 M group, *n* = 126, receiving 126 embryos), and (d) patients receiving day 5 SET with a blastocyst selected by morphology plus Eeva™ (SET-5 ME group, *n* = 48, receiving 48 embryos). Overall, a clinical pregnancy rate of 49.1%, implantation rate of 40%, and ongoing pregnancy rate of 43.6% were observed. The implantation rate was significantly higher in DET-3 ME group than in DET-3 M group (44.8% vs. 30.2%, *p* < 0.02), whereas it was comparable in groups DET-3 ME, SET-5 M and SET-5 ME. Differently, the ultrasound-verified clinical pregnancy rate and the ongoing pregnancy rate at 12 weeks did not significantly differ in all four groups. Overall, our findings suggest that Eeva™ algorhythm can improve embryo selection accuracy of standard morphology when ET on day 3 is scheduled, leading to a higher implantation rate, but its impact on ongoing pregnancy and live birth needs to be further clarified.

## Introduction

Selecting the best embryo(s) to be transferred in uteri in the same cycle or cryopreserved for a delayed transfer is a key issue in human IVF. In the last decades, embryo morphology was widely used for detecting embryo competence, and several morphological scores applying to cleavage stage (day 2 or 3) embryos were proposed, none of them, however, being adopted as a worldwide-accepted standard. The strategy of transferring a single embryo to avoid twin pregnancy was progressively adopted in the last years, and extended culture to the blastocyst stage, although rather complicated and time-consuming, became quite popular, raising the need for blastocyst morphological scores; also in this case, however, a thoroughly reliable method to select the best blastocyst was not yet found.

Time-lapse embryo culture technology (TLT) is a rather recent approach to embryo selection. It allows the continuous, dynamic assessment of embryo morphological changes without the need to remove embryos from the incubator, thus limiting potentially detrimental effects of changes in culture conditions [8]. To date, however, only some studies reported higher clinical and ongoing pregnancy rates using embryo selection by TLT vs. the classical morphological embryo selection [12], whereas other prospective randomized trials did not find any improvement in IVF outcome with TLT [5, 9, 10]. To date, a convincing, final demonstration that TLT can improve IVF results in terms of live births is still lacking.

Early Embryo Viability Assessment (Eeva™) is an algorhythm that applyes to a specific type of TLT system; it was designed to predict blastocyst formation on the basis of morphokinetic parameters observed in the first three days of embryo growth. Eeva™ was aimed at indicating on day 3 which embryos are more likely to develop into viable blastocysts, and gives the potential advantage to select the most competent embryos on day 3 without the need to extend culture till day 5, thus saving time and resources. Until now, however, only a few studies assessing the impact of Eeva™ on cleaving embryo selection were performed: a multicenter study concluded that the combination of standard morphology plus Eeva™ was more effective than standard morphology alone in identifying embryos able to reach the blastocyst stage [2]; other studies reported that Eeva™ application improved the possibility to select embryos and increased implantation and pregnancy rates [1, 16]. On the other side, however, some authors reported no significant differences in pregnancy rates when Eeva™ was used in association with standard morphology vs. morphology alone on day 3 [9] or vs. morphological selection alone on day 5 [7, 18].

In the present study we aimed at retrospectively assessing in a very homogeneously selected group of IVF patients whether the addition of Eeva™ to standard morphology increased the accuracy of embryo selection in case of double embryo transfer (DET) on day 3 or single embryo transfer (SET) on day 5.

## Materials and methods

### Patients

The study was designed as a retrospective analysis of IVF cycles performed at our IVF Unit between March 2016 and July 2018. In order to minimize the risk of interference by patient-related characteristics, we included in the study a very homogenous group of carefully selected patients chosen with the following, strict criteria: (a) age 28–40 years; (b) body mass index (BMI) 18–25); (c) ovarian reserve markers predicting a normal response to gonadotropins (serum day 3 FSH < 12 IU/l, AMH 1.2–5 ng/ml, antral follicle count (AFC) 8–15); (d) verified normal response to controlled ovarian stimulation (COS) performed by gonadotropin-releasing hormone (GnRH)-agonist ″long″ protocol plus recombinant FSH, with at least 6 retrieved oocytes; (e) easy transfer of two embryos on day 3 (DET) or (in case of at least 4 good-scoring embryos on day 3) of a single blastocyst on day 5 (SET).

The study was carried out in accordance to the Declaration of Helsinki and was authorized as an observational study by the local Ethical Committee. A signed, written consent was retrospectively obtained from all patients accepting their data being included in the analysis.

### Controlled ovarian stimulation (COS) and oocyte retrieval

All included patients underwent COS using the gonadotropin-releasing hormone (GnRH)-agonist ″long″ protocol with recombinant FSH (Gonal-F®, Merck, Darmstadt, Germany) at individually tailored starting daily dose (125–250 IU/day, subcutaneously). Follicular growth was monitored by transvaginal US examination plus serial measurements of circulating estradiol (E2), performed every second day from stimulation day 7, and adjusting FSH dose accordingly. When at least two follicles reached 18 mm mean diameter, with appropriate E2 levels, a single subcutaneous injection of 10,000 IU hCG (Gonasi HP, IBSA, Pambio Noranco, Switzerland) was administered to trigger ovulation. US-guided oocyte retrieval (OPU) was performed 35–37 h later under local anesthesia (paracervical block). The aspirated follicular fluid was immediately observed under stereomicroscope to retrieve the corresponding oocyte, that was then washed in buffered medium and incubated in controlled atmosphere until fertilization procedure.

### Preparation of semen samples and in vitro fertilization

Semen samples were examined to assess sperm concentration, motility, and morphology according to the World Health Organization guidelines, and were then prepared by density gradient centrifugation in order to select normally motile, morphologically normal spermatozoa. Conventional IVF or ICSI injection was performed on all available oocytes within 4 h after OPU, and the occurrence of normal fertilization was assessed after 16–18 h of incubation in controlled atmosphere by evaluating the presence of two pronuclei (2PN) and the extrusion of the second polar body.

### Embryo selection and transfer

Fertilized oocytes were placed in pools in a 4-wells dish (Thermo Scientific, Denmark), and were cultured in pre-equilibrated Cleavage medium (Cook) overlain with mineral oil, using the same tri-gas box incubators (Panasonic) containing an atmosphere of 5% O2 and 6–7% CO2, balanced with N2. A medium refresh step on day 3 was performed.

When only the classical morphological evaluation was applied, the Integrated Morphology Cleavage Score (IMCS) was used. IMCS is the only score constructed to be evidence-based, as it was obtained comparing implanted embryos vs. non-implanted embryos in a rather large number of IVF cycles ending in double embryo transfer (DET) [6]. Due to its peculiar characteristics, IMCS has been incorporated in a complex prediction model for IVF outcome, proven to predict live birth with a remarkably good accuracy [14]. After IMCS-based morphological selection, either a double embryo transfer (DET) on day 3 or a single blastocyst transfer (SET) on day 5 were performed. SET was chosen when at least 4 good scoring (> 8/10 at IMCS) embryos were available on day 3; in this case, the blastocyst morphological selection on day 5 was performed as previously described [15] and the transferred embryo was chosen taking into account both the IMCS score and the blastocyst morphological score.

When the Eeva™ system was added to standard morphology to assess embryo competence, embryos were cultured in microwells of the Eeva™ dish (12 wells/dish; one embryo/well), whose format allows following each embryo individually even if all embryos share a common 100 μL drop of medium (group culture). Embryos were cultured in pre-equilibrated Cleavage medium (Cook, Ireland) overlaid with mineral oil. Eeva™ microscopes were housed in tri-gas box incubators (Panasonic) containing an atmosphere of 5% O2 and 6–7% CO2, balanced with N2. A medium refresh step on day 3 was performed using a new culture dish with pre-equilibrated Blastocyst medium (Cook, Ireland) where embryos were placed. Actually Eeva™ culture dishes have 12 microwells under a single drop of medium: each well is identified by a letter (A, B, C, D) and a number (1, 2, 3): on day 3, embryos were moved from one dish to the other keeping the same microwell order (A1 to A1 in the new dish, etc.): This allowed to track individual embryo development and accurately correlate Eeva™ score on day 3 with embryo assessment on day 5. Dark field images were acquired every 5 min from the time of culture start until ET, cryopreservation or discharge; a video frame corresponded to 5 min in culture. All embryos were likewise assessed by bright field microscopy on day 3 to manually insert blastomere number, allowing calculating Eeva™ score prior to ET. Eeva™ algorhythm generated a calculation of High or Low probability of blastocyst formation based on kinetic growth parameters: the P2 value (time between first and second mitosis; P2 “high” range: 9.33–11.47 h) and the P3 value (time between second and third mitosis; P3 “high” range: 0.00–1.73 h). After morphology plus Eeva™-based embryo selection, either two embryos with “high” probability of blastocyst formation (DET) or one blastocyst on day 5 (SET) were transferred. In case of day 5 ET, the transferred embryo was chosen taking into account both the Eeva™ indication and the blastocyst morphological score; in detail, Eeva™ rating was used to chose between blastocysts with a similar morphological score.

The patients included in the study belonged to four groups: (a) those receiving day 3 DET with embryos selected by IMCS (DET-3 M group, *n* = 106, receiving 212 embryos), (b) those receiving day 3 DET with embryos selected by morphology plus Eeva™ (DET-3 ME group, *n* = 48, receiving 96 embryos), (c) those receiving day 5 SET with a blastocyst selected by conventional morphology (IMCS and blastocyst score) (SET-5 M group, *n* = 126, receiving 126 embryos), and (d) those receiving day 5 SET with a blastocyst selected by morphology plus Eeva™ (SET-5 ME group, n = 48, receiving 48 embryos).

### Statistical analysis

Comparison among groups was performed using SAS (SAS Institute, Cary, NC) software package, using the unpaired t-test for continuous variables (shown as mean ± SD) and the Fisher exact test for categorical variables (shown as percentage). All statistical tests were two-sided and a *P* value of 0.05 or less was considered statistically significant.

## Results

Among a total number of 1178 couples that completed IVF cycle in the study time period, 356 matched the inclusion criteria, and 328 of them authorized the inclusion of their data in the analysis. The clinical characteristics of the enrolled patients and the outcome of their IVF cycles are summarized in Tables 1 and 2, respectively.

**Table 1:** Clinical data of enrolled patients according to the embryo selection method

	All	DET-3 M	DET-3 ME	SET-5 M	SET-5 ME	p
	(*n* = 328)	(*n* = 106)	(n = 48)	(n = 126)	(n = 48)	
Age (years)	34.6 ± 3.0	34.7 ± 3.4	34.8 ± 3.0	34.2 ± 2.9	34.9 ± 1.9	*ns*
BMI (kg/m^2^)	23.6 ± 3.9	23.1 ± 3.6	23.2 ± 4.2	24.1 ± 4.2	24.6 ± 3.5	*ns*
Day 3 FSH (IU/I)	6.9 ± 1.5	7.1 ± 1.7	6.7 ± 1.3	6.7 ± 1.3	6.9 ± 1.8	*ns*
AMH (ng/ml)	3.7 ± 2.5	3.3 ± 1.9	3.0 ± 1.8	4.6 ± 3.2	3.6 ± 2.0	*ns*
AFC	15.4 ± 7.3	14.3 ± 6.0	15.4 ± 9.4	16.9 ± 8.2	15.2 ± 5.5	*ns*
Total exogenous FSH (IU)	2083.7 ± 823.7	2245.2 ± 940.7	2112.0 ± 841.0	1902.2 ± 671.2	2026.2 ± 736.7	*ns*
Peak E2 (pg/ml)	2437.2 ± 1097.9	1933.9 ± 867	1672.9 ± 1178.3	2211.3 ± 1171.1	2436.3 ± 1317.3	*ns*
OSI	6.6 ± 3.8	5.3 ± 3.0	5.5 ± 3.6	7.9 ± 3.6	8.3 ± 5.2	< 0.05*
Endometrial thickness (mm)	10.4 ± 2.0	10.2 ± 1.8	10.0 ± 2.2	10.7 ± 2.2	10.6 ± 1.7	*ns*
Retrieved oocytes	11.6 ± 4.0	10.2 ± 3.0	9.5 ± 2.6	13.4 ± 4.2	13.8 ± 4.9	< 0.05*
Mature (MII) oocytes (%)	83.5 ± 15.2	85.6 ± 12.6	78.1 ± 19.5	81.8 ± 15.6	89.1 ± 12.6	*ns*
Insemination technique (%)						*ns*
IVF	24 (40/164)	26 (14/53)	25 (6/24)	25 (16/63)	17 (4/24)	
ICSI	76 (124/164)	74 (39/53)	75 (18/24)	75 (47/63)	83 (20/24)	
Fertilized (2PN) oocytes (%)	74.4 ± 17.6	69.8 ± 18.6	72.8 ± 18.3	79.7 ± 15.2	76.7 ± 16.6	*ns*
Cleaved embryos (%)	97.3 ± 7.9	96.8 ± 7.7	96.3 ± 11.6	98.0 ± 6.3	98.2 ± 7.1	*ns*

DET-3 M = patients receiving two day 3 embryos selected by IMCS; DET-3 ME = patients receiving two day 3 embryos selected by standard morphology plus Eeva™ score; SET-5 M = patients receiving one day 5 blastocyst selected by IMCS plus blastocyst morphological score; SET-5 ME = patients receiving one day 5 blastocyst selected by blastocyst morphological score plus Eeva™. AFC = antral follicle count. OSI = ovarian sensitivity index (retrieved oocytes × 1000 / total FSH dose). Data are expressed as mean ± SD or as absolute value and percentage. * Significance is referred to the comparison of both SET subgroups (SET-5 M and SET-5 ME) vs. both DET subgroups (DET-3 M and DET-3 ME)

**Table 2:** Clinical outcome of IVF according to the embryo selection method

	All (n = 328)	Day 3 M	Day 3E	Day 5MM	Day 5 EM	p
		(n = 106)	(n = 48)	(n = 126)	(n = 48)	
Transferred embryos	482	212	96	126	48	
Clinical pregnancy rate (%)	49.1 (161/328)	45.3 (48/106)	56.2 (27/48)	49.2 (62/126)	50.0 (24/48)	*ns*
Implantation rate (%)	40.0 (193/482)	30.2 (64/212)	44.8 (43/96)	49.2 (62/126)	50.0 (24/48)	< 0.02*
Twin pregnancy rate (%)	19.8 (32/161)	33.3 (16/48)	59.2 (16/27)	0/62	0/24	
Ongoing pregnancy rate (%)	43.6 (143/328)	39.6 (42/106)	50.0 (24/48)	43.6 (55/126)	45.8 (22/48)	*ns*

DET-3 M = patients receiving two day 3 embryos selected by IMCS; DET-3 ME = patients receiving two day 3 embryos selected by standard morphology plus Eeva™ score; SET-5 M = patients receiving one day 5 blastocyst selected by IMCS plus blastocyst morphological score; SET-5 ME = patients receiving one day 5 blastocyst selected by blastocyst morphological score plus Eeva™. Data are expressed as absolute value and percentage. * Significance is referred to the comparison of DET-3 M vs. DET-3 ME, SET-5 M and SET-5 ME

All ETs were performed by experienced operators (AR, AC, GG, LDP) using the soft catheter Sydney Guardia (Cook, Australia) under transvaginal ultrasound guidance, applying the method that was previously published by our group [13]. No ET resulted to be difficult or forced to change catheter and to repeat the procedure.

Both subgroups of patients who received SET on day 5 had significantly more retrieved oocytes and higher ovarian responsiveness to COS (ratio between total FSH dose and retrieved oocytes, OSI) than the two subgroups that had DET on day 3 (Table 1). This is due to the fact that blastocyst transfer was performed only in patients with at least four good scored embryos on day 3, whereas all others patients received DET on day 3.

Overall, 1818 embryos were obtained in 328 IVF cycles; 482 embryos were transferred in uteri, and 193 implanted (overall implantation rate: 40%) originating 161 US-verified clinical pregnancies (overall clinical pregnancy rate: 49.1%). Thirty-two pregnancies were twin pregnancies, leading to an overall twinning rate of 19.8%, which is close to the average IVF twinning rate in Italy; however, single blastocyst transfer on day 5 never originated a twin pregnancy, whereas DET on day 3 obtained a very high twinning rate (33.3% in the DET-3 M group, 59.2% in the DET-3 ME group) (Table 2).

The clinical pregnancy rate was comparable in the four subgroups, with slight differences (Table 2). The implantation rate observed in DET-3 ME group was significantly higher than the one of DET-3 M group (44.8% vs. 30.2%, *p* < 0.02), whereas it was comparable to those of groups SET-5 M and SET-5 ME (Table 2). Eighteen pregnancies underwent a spontaneous miscarriage in the first trimester, and finally the ongoing pregnancy rate at 12 weeks gestational age was similar in the four subgroups, without any significant difference (Table 2).

## Discussion

The selection of embryos having the highest competence for pregnancy and live birth has been based for years on a single observation by inverted light microscopy performed on day 2, 3 or 5 of embryo culture. Repeated observations, although likely to give better insights about embryo competence, were used with caution because even a short exposure of embryos to suboptimal conditions outside the controlled environment of the incubator was thought to potentially affect the implantation potential.

The introduction of time-lapse technology (TLT) into the clinical practice has allowed providing a continuous surveillance of embryo growth, while maintaining stable culture conditions; moreover, the recording of previously unknown kinetic parameters of embryo development has provided new embryo-related variables available for analysis [8]. TLT was claimed to have the potential to improve embryo selection capability and, as a consequence, IVF outcome; indeed some studies reported higher pregnancy rates using TLT (reviewed in [12]), but others could not confirm this finding as they failed to observe any improvement of IVF results vs. the standard morphological embryo selection [5, 9, 10].

So far, none of the studies comparing TLT to the single observation, morphological embryo assessment used the evidence-based score named IMCS as a reference [6]. The difference between IMCS and the other scoring methods is that IMCS was constructed comparing the morphology of surely implanted embryos (dizygotic twin pregnancies after DET) vs. surely non-implanted embryos (no pregnancy after DET), and was therefore based on the evidence of implantation and clinical pregnancy. Indeed IMCS is the morphological score that was incorporated into a complex prediction model for IVF outcome, recently shown to predict live birth with a remarkably good precision [14]. Actually the present study is the first comparing embryo selection performed by an evidence-based morphological score vs. TLT.

In our study we aimed at assessing the impact of the adjunctive use of the Early Embryo Viability Assessment (Eeva™), an algorhythm for automatic embryo scoring at the cleavage stage, on the embryo selection process performed using conventional morphology. The possibility to predict the development of embryos observed in the first days of growth to the blastocyst stage, a concept underlying Eeva™ test, was previously demonstrated [3, 11, 17], and Eeva™ was found to be more reliable than a panel of embryologists with diverse experience in assessing embryo potential to evolve to blastocyst [4]. The clinical application of Eeva™ was already tested in a few studies, but conflicting results were obtained: an improved possibility of identification of cleaving embryos prone to reach the blastocyst stage was shown in a multicenter study [2], a positive effect of Eeva™ application on implantation and pregnancy rates was reported in other studies [1, 16], but some authors failed to observe any significant difference in pregnancy rates when Eeva™ was used in association with standard morphology vs. morphology alone performed on day 3 [9] or day 5 [7, 18].

We performed the present analysis on a very homogeneous patients’ population, that was selected using very strict inclusion criteria; this strategy allowed obtaining four groups of patients with very similar clinical characteristics. The potentially confounding variable of the day chosen for ET (day 3 vs. day 5) was accounted for including in the study a subgroup in which Eeva™ was performed on day 3, but a single embryo was transferred on day 5, after a selection process that considered together Eeva™ results and blastocyst morphology.

Overall, we observed that when two embryos were selected using morphology plus Eeva™ and transferred on day 3 (DET-3 ME group), the implantation rate was significantly higher than when the IMCS alone was used (DET-3 M group); interestingly enough, the implantation rate of Eeva™-selected embryos on day 3 was comparable to the one of blastocysts morphologically selected and transferred on day 5 (SET-5 M and SET-5 ME groups). On one side this led to an unacceptably high twinning rate in the DET-3 ME group - suggesting that when Eeva™ is used to select embryos, only one embryo should be transferred in uteri – on the other side it demonstrated that Eeva™ has a remarkable efficacy in identifying which day 3 embryos have the best chance of development to blastocyst and implantation.

The positive effect of Eeva™ addition to classical morphology on the accuracy of embryo selection, however, was lost when embryo culture was prolonged to day 5; in fact, comparable implantation rates were obtained after single blastocyst transfer regardless Eeva™ was considered or not to chose the embryo to transfer. To this purpose it should be remarked that Eeva™ was constructed for use on day 3, and probably considering its results for the blastocyst selection process on day 5 represents an improper use.

Despite the described differences in the implantation rate, in our study both the clinical pregnancy rate and the ongoing pregnancy rate at 12 weeks were not significantly different in the four groups. This may be likely due to the relative low number of observations in some groups, but it may be noticed that even other authors reported that Eeva™ was ineffective in increasing the ongoing pregnancy rate when compared to a standard morphological score [7, 9]. Also a study showing an increased pregnancy rate with Eeva™, unfortunately did not report about the ongoing pregnancy rate [1].

A limitation of our study, besides its retrospective nature, was that embryos were cultured in different incubators (Eeva™ vs. low oxygen tension incubators), culture dishes and volumes, and the influence of the culture conditions could not be clearly distinguished from the effect of embryo selection strategy. This confounder could not be eliminated because Eeva™ system cannot be housed in any incubator, but requires a specific model.

We are aware that our results may not be considered conclusive and should be verified on a larger scale and/or in properly weighted prospective trials. However, with all the above limitations, our findings suggest that Eeva™ algorhythm could be useful in improving the embryo selection accuracy of standard morphology when ET on day 3 is scheduled. Further, Eeva™ could be quite useful when applied to perform a SET on day 3, as according to our findings it could obtain clinical results comparable to the more time-demanding SET on day 5, after blastocyst culture.

## Data Availability

The study is a retrospective analysis of IVF cycles performed at our IVF Unit between March 2016 and July 2018. Data are stored in our institutional archive.

## Abbreviations

AFC: antral follicle count
AMH: anti-Mullerian hormone
BMI: body mass index
COS: controlled ovarian stimulation
DET: double embryo transfer
Eeva™: Early Embryo Viability Assessment
ET: embryo transfer
IMCS: Integrated Morphology Cleavage Score
OPU: US-guided oocyte retrieval (ovum pick-up)
SET: single embryo transfer
TLT: Time-lapse embryo culture technology
